# Development and Application of SSR Markers for *Aquilaria sinensis* on the Basis of Whole-Genome Resequencing Data

**DOI:** 10.3390/plants14091323

**Published:** 2025-04-27

**Authors:** Yu Chen, Kunlin Wu, Jieru Xu, Shenghe Zhao, Zhihua Tu, Dandan Rao, Beibei Chen, Nanbo Jiao, Jinhui Chen, Xiaona Dong

**Affiliations:** 1 Hainan Academy of Forestry (Hainan Academy of Mangrove), Haikou 571100, China; cfstuchen@126.com (Y.C.); raodand@163.com (D.R.); 2 School of Breeding and Multiplication (Sanya Institute of Breeding and Multiplication)/School of Tropical Agriculture and Forestry, Hainan University, Sanya 572019, China; kunlinwu@hainanu.edu.cn (K.W.); jieruxu@hainanu.edu.cn (J.X.); zhaoshenghe@hainanu.edu.cn (S.Z.); tuzhihua@hainanu.edu.cn (Z.T.); jiaonanbo@id-biotech.com (N.J.); 3 Key Laboratory of Tropical Forestry Resources Monitoring and Application of Hainan Province, Haikou 571100, China; 4 College of Coastal Agricultural Sciences, Guangdong Ocean University, Zhanjiang 524088, China; beibeichenk@outlook.com

**Keywords:** whole-genome resequencing (WGR), simple sequence repeat (SSR), *Aquilaria sinensis*, genetic variation, polymorphism

## Abstract

*Aquilaria sinensis* (Lour.) Spreng. is an economically important tree specie that produces agarwood, a valuable medicinal and aromatic resin, when injured. However, its large-scale cultivation has led to confusion regarding its resources and genetic backgrounds, hindering the conservation and management of *A. sinensis* accessions. This study systematically developed and validated simple sequence repeat (SSR) molecular markers by using whole-genome resequencing (WGR) data from 60 *A. sinensis* accessions to elucidate their genetic diversity and population structure. A total of 56,657 SSR sequences (24,430 loci) were identified, which were dominated with dinucleotide repeat motifs (73.59%). After stringent quality control, 46 high-quality SSR loci were obtained, and 93 primer pairs were designed for amplification validation. Ultimately, 20 primer pairs with stable amplification and high polymorphism were selected, of which 11 exhibited high polymorphism (polymorphic information content: 0.554–0.688). These 20 primer pairs identified a total of 121 alleles, with an average of 6 alleles per locus. These primers successfully classified 149 *A. sinensis* accessions into three subpopulations, achieving a discrimination rate of 95.97%. The analysis of molecular variance revealed that genetic variation within the individuals accounted for 84% of the total variation. This study establishes a rapid and efficient SSR-based method by leveraging resequencing data for large-scale marker discovery in *A. sinensis*. It further provides a robust technical framework for the conservation and sustainable utilization of this valuable species.

## 1. Introduction

*Aquilaria sinensis* (Lour.) Spreng., a tree species of the *Thymelaeaceae* family, is renowned for its ability to produce agarwood, a resinous substance formed in response to external injury and fungal infection [1,2]. *A. sinensis* produces terminal or axillary umbellate inflorescences with bisexual flowers. This species exhibits self-incompatibility and is primarily pollinated by noctuids, pyralids, and geometrids [3,4]. Seed dispersal is primarily mediated by hornets [4].

Agarwood is highly valued not only for its medicinal properties, but also as a precious incense in religious and cultural practices, with a long history of utilization in regions such as India, the Middle East, China, and Japan [5]. Pharmacological studies have demonstrated that bioactive components in agarwood, including sesquiterpenes and phenylethyl chromone derivatives, exhibit anti-inflammatory, sedative, antioxidant, antimicrobial, and antitumor activities [6,7,8,9,10,11]. By 2013, the global agarwood market had reached an estimated value of USD 6–8 billion, with the market continuing to expand rapidly [12]. In China, *A. sinensis* was historically distributed across multiple southern provinces, including Hainan, Guangdong, Guangxi, and Yunnan. However, healthy *A. sinensis* trees do not produce agarwood, and the overexploitation of wild populations has led to their sharp decline [13]. Although artificial cultivation and agarwood induction techniques (e.g., mechanical drilling, electrical stimulation, and fungal inoculation) have been widely adapted, challenges such as unclear accession origins, ambiguous genetic backgrounds, and cultivar misidentification severely hinder the sustainable utilization of *A. sinensis* and the efficient production of agarwood [14,15,16]. Therefore, developing a rapid and accurate accession identification method is critical for the conservation and genetic improvement of this species.

Simple sequence repeat (SSR) markers, also known as microsatellites, consist of motifs of tandem repeats of 1–6 nucleotides. Owing to their codominant inheritance, high polymorphism, genome-wide distribution, reproducibility, and technical simplicity, SSR markers have been extensively applied in genetic diversity analysis, germplasm identification, genetic linkage mapping, and marker-assisted breeding [17,18,19]. For instance, SSR marker development in rice and wheat has significantly advanced the construction of genetic maps and trait-associated gene localization [20,21]. However, SSR-based studies on *A. sinensis* are still limited. Zou et al. used SSR and sequence-related amplified polymorphism (SRAP) markers to analyze the genetic diversity of 112 agarwood accessions and classified them into five geographically distinct groups that were consistent with their origins [22]. Xu et al. further identified 407 SSR loci within 92 candidate genes associated with agarwood formation and utilized 16 polymorphic primers to investigate the genetic diversity and population structure of 179 *A. sinensis* accessions [13]. Despite these developments, the lack of genome-wide SSR marker development has restricted the comprehensive exploration and utilization of *A. sinensis* genetic resources.

Recent advancements in whole-genome resequencing (WGR) technology, coupled with reduced costs, have revolutionized molecular marker development and genetic breeding in plants. High-throughput sequencing enables the rapid identification of genetic markers, including single nucleotide polymorphisms (SNPs), insertions/deletions (InDels), and structural variations (SVs). It also integrates functional genomics to elucidate the genetic basis of traits, thereby enhancing the efficiency of the marker-assisted selection. As of 2023, WGR has been applied to 187 terrestrial plant species, encompassing 54,413 accessions of cereals, legumes, vegetables, and other crops, and has resulted in successfully mapping the loci associated with yield, stress tolerance, and quality traits [23]. These studies have shown that WGR not only facilitates the development of efficient markers, but also accelerates the genetic improvement of complex traits through integration with transcriptomic and phenotypic data.

In this study, we developed genome-wide SSR markers for *A. sinensis* by using WGR data, and evaluated their polymorphism and application potential. A total of 56,657 SSR loci were identified from 168,459 sequences, from which 46 high-quality SSR loci were selected after stringent screening. Primers designed for these loci were preliminarily validated using eight DNA samples, which yielded 20 pairs with stable and reproducible amplification for subsequent genetic diversity assessment. By establishing a high-density SSR marker system, this research provides an essential tool for accession identification, genetic diversity analysis, and the molecular breeding of *A. sinensis* while laying a foundation for elucidating the genetic mechanisms underlying the agarwood formation.

## 2. Results

### 2.1. SSR Locus Characteristics

A total of 56,657 SSR sequences were identified from 168,459 sequences, which comprised 4747 mononucleotide repeats (8.38%), 41,695 dinucleotide repeats (73.59%), 9550 trinucleotide repeats (16.86%), 644 tetranucleotide repeats (1.14%), 13 pentanucleotide repeats (0.02%), and 8 hexanucleotide repeats (0.01%) (Table 1). After accounting for complementary base pairing, 86 distinct motifs were identified across 149 *A. sinensis* accessions. Tetranucleotide motifs were the most diverse (34 types), followed by trinucleotide motifs (28 types), while mono-, di-, penta-, and hexanucleotide motifs contained 2, 8, 8, and 6 types, respectively (Table S1). Among the mononucleotide repeats, A/T motifs were the most abundant (3013; 63.47%), followed by C/G motifs (1731; 36.53%). AT/AT motifs (15,194; 36.44%), followed by TA/TA motifs, were the most dominant dinucleotide repeats (12,840; 30.80%). Among the trinucleotide repeats, TAT/ATA motifs were predominant (3007; 31.49%), followed by AAT/ATT (2789; 29.20%) and TTA/TAA (2749; 28.79%). The highest frequencies were observed for TATG/CATA motifs (228; 35.40%) and ATAC/GTAT (135; 20.96%) tetranucleotide repeats, whereas the penta- and hexanucleotide motifs exhibited limited representation and dispersed distributions (Table S1).

The genotypic frequencies of the SSR loci across the 149 *A. sinensis* accessions were calculated. The most frequent SSR alleles ranged from 0.15 to 0.87, followed by secondary (0.08–0.47) and tertiary alleles (0.00–0.28) (Table S2). Notably, the SSR locus Scaffold_5532_109254228 (with a CTT repeat motif) showed equal allele frequencies for the most common and the second-most common alleles (both at 0.15), with a slight difference from the third allele (frequency of 0.13). Similar patterns were observed for the loci Scaffold_5532_8308141, Scaffold_8152_16679434, and Scaffold_3585_64368533 (Table S2). All the other SSR loci displayed a dominance across the genotypes within the population. These SSR profiles provide a foundation for marker-assisted selection and breeding strategies in *A. sinensis.*

### 2.2. SSR Primer Development

After collapsing and statistically analyzing SSR loci from identical genomic regions, 51,910 SSR sequences were found to originate from 24,430 SSR loci. Thus, 24,430 SSR loci were identified in *A. sinensis* using the population genome resequencing-derived VCF file. After the removal of the mononucleotide repeats, 22,486 SSR loci remained. The further exclusion of compound loci (containing multiple SSRs within 100 bp) resulted in 13,560 candidate loci.

A total of 152 SSR loci passed quality control. After excluding the loci lacking secondary common genotypes (defined as the second-most frequent genotype in the population), 46 high-confidence SSR loci were retained.

Three primer pairs were designed for each of the 46 SSR loci. Primers with short target fragments, ≥4 consecutive nucleotide repeats, or extreme GC contents were discarded; this yielded 93 candidate primer pairs. To identify polymorphic SSR loci, primers were initially screened (Table S3) using three *A. sinensis* DNA samples.

Primers that failed to amplify ≥3 distinct bands, that produced ambiguous peaks, or that showed amplification failures were discarded. A secondary screening (Table S4) was conducted by using eight randomly selected *A. sinensis* DNA samples that confirmed primers that yielded ≥3 reproducible bands and had polymorphism information content (*PIC*) > 0.30. Ultimately, 20 polymorphic SSR primer pairs were selected for genetic diversity analysis. Among these, trinucleotide repeats dominated (15 pairs), followed by dinucleotide repeats; only AquSSR34 represented a tetranucleotide repeat (Table 2).

### 2.3. SSR Polymorphism Evaluation

The polymorphism of 20 SSR loci was evaluated across 149 *A. sinensis* accessions on several parameters, which we briefly describe below. A total of 121 alleles were detected, where the number of observed alleles (*Na*) per locus ranged from 3 to 12 (mean: 6.050). AquSSR07, AquSSR34, and AquSSR89 exhibited the lowest *Na* (3 alleles each), while AquSSR29 showed the highest *Na* (12 alleles; Table 3). The effective number of alleles (*Ne*) ranged from 1.256 (AquSSR14) to 3.751 (AquSSR10), with a mean of 2.511. Notably, *Ne* values were consistently lower than *Na* across all the loci, which indicated an uneven allele distribution in the population (Table 3).

Further, the observed heterozygosity (*Ho*) ranged from 0.181 (AquSSR22) to 0.932 (AquSSR94), with a mean of 0.495. The expected heterozygosity (*He*) varied between 0.204 (AquSSR14) and 0.733 (AquSSR10), averaging 0.557 (Table 3). Furthermore, the Shannon’s information index (*I*) values ranged between 0.478 (AquSSR14) and 1.457 (AquSSR40), with an average of 1.063 (Table 3). These results indicate moderate levels of genetic diversity within the studied population.

Moreover, the polymorphism information content (*PIC*) values ranged from 0.196 (AquSSR14) to 0.688 (AquSSR10), with a mean of 0.507. Based on *PIC* classification thresholds (low: *PIC* < 0.25; moderate: 0.25 ≤ *PIC* < 0.50; high: *PIC* ≥ 0.50), two loci (AquSSR14, AquSSR34) were categorized as exhibiting low polymorphism, seven showed moderate polymorphism, and eleven displayed high polymorphism. AquSSR10 demonstrated the highest *PIC* value (0.688), underscoring its utility in genetic analyses (Table 3).

### 2.4. Genetic Diversity Analysis

#### 2.4.1. Clustering Analysis

**Figure 1:**
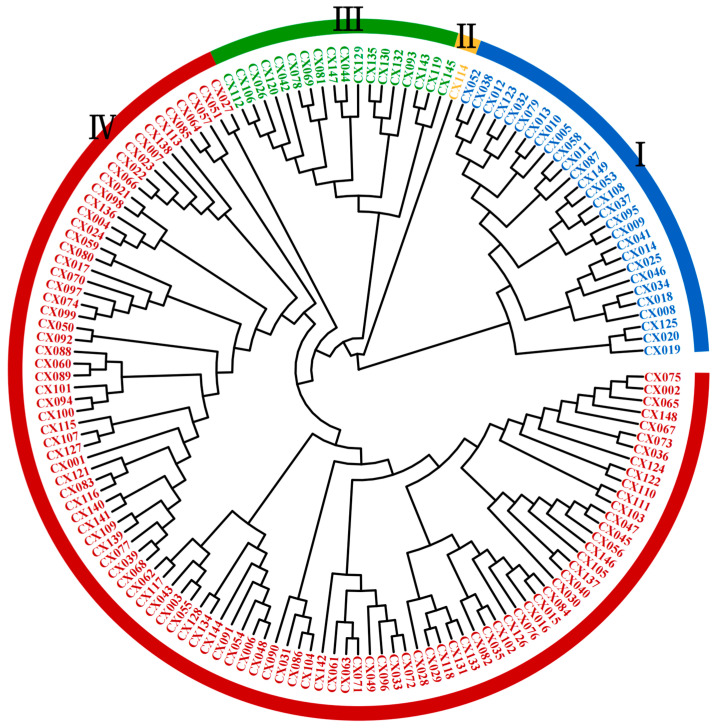
Cluster analysis of 149 *A. sinensis* accessions based on *Nei’s* genetic distance. Groups I–IV are represented by blue, yellow, green, and red.

UPGMA clustering based on *Nei’s* genetic distance classified the 149 *A. sinensis* accessions into four major groups, which are represented by distinct colors in Figure 1. Group I comprised 28 accessions (18.79% of the total), namely, CX052, CX038, CX012, CX123, CX032, CX079, CX013, CX010, CX005, CX058, CX011, CX087, CX149, CX053, CX108, CX037, CX095, CX009, CX041, CX014, CX025, CX046, CX034, CX018, CX008, CX125, CX020, and CX019. Group II contained a single accession (CX114, 0.67%), exhibiting significant genetic divergence from the others. Group III comprised 18 accessions (12.08%), that is, CX112, CX106, CX026, CX120, CX042, CX078, CX069, CX081, CX147, CX044, CX129, CX135, CX130, CX132, CX093, CX143, CX119, and CX145. The remaining 102 accessions (68.46%) formed Group IV. The distinct clustering of CX114 suggests its potential value for its inclusion in core germplasm repositories (Figure 1).

#### 2.4.2. Population Genetic Structure

**Figure 2:**
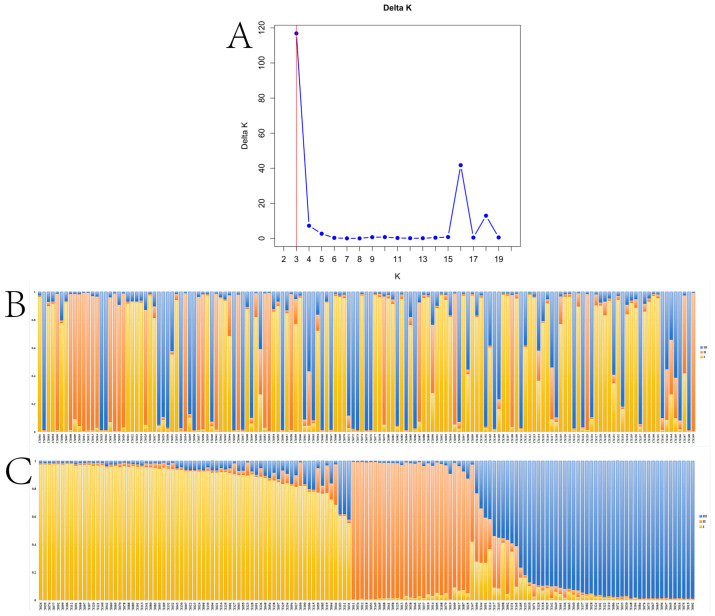
Population structure analysis of 149 *A. sinensis* accessions based on Structure; (**A**) ΔK estimation plot (the Structure analysis commenced with the assumption that the population comprises multiple subpopulations (k = x). The simulation algorithm was employed to ascertain the most appropriate classification method to be obtained in the scenario where ΔK attains its maximum value.); (**B**) predicted population structure of 149 accessions; (**C**) three genetic clusters identified with Structure analysis. Clusters I-III are represented by yellow, orange, and blue.

Next, we analyzed the genetic structure of the 149 accessions with the help of Structure, which revealed clear genetic differentiation and distinct clustering patterns among different groups. Further analysis using Structure2.3.4 revealed optimal population subdivision at K = 3 (the meaning of the K-value is the number of clusters by which the group is intended to be divided; ΔK = 116.890; Figure 2, Table S5).

#### 2.4.3. Population Genetic Diversity Analysis

Genetic diversity indices for the three clusters divided using population genetic structure analysis were calculated with GenAlex6.5 (Table 4 and Table S5): *Ho* ranged from 0.471 to 0.561 (mean: 0.505), while *He* varied between 0.470 and 0.603 (mean: 0.526). Cluster II exhibited the highest Ho and He values. Further, *F* values ranged from −0.002 to 0.063, with Cluster III showing negative *F* values, which indicated a higher heterozygosity. *I* averaged 0.975 (range: 0.851–1.141), with Cluster II displaying the highest genetic differentiation. Overall, moderate genetic diversity was observed across clusters, with Cluster II exhibiting the greatest divergence.

An analysis of molecular variance (AMOVA) revealed 11% of genetic variation among populations and 89% within populations (Table 5). Pairwise *Fst* values (0.051–0.085; Table 6) indicated low genetic differentiation (*Fst* < 0.15) among the three clusters.

#### 2.4.4. Fingerprint Profiling

A binary matrix (0/1) was generated on the basis of the presence/absence of SSR amplification bands across the 20 loci (126 alleles). All the accessions, except CX002/CX075, CX015/CX084, and CX103/CX111, were unambiguously distinguished, which achieved a discrimination rate of 95.97% (Figure S1).

## 3. Discussion

SSRs, also known as microsatellites or short tandem repeats (STRs), consist of 1–6 nt repeat units that are widely distributed across a plant genome. Due to their high informativeness, polymorphism, codominant inheritance, and reproducibility, SSRs are widely used for studying genetic diversity, constructing genetic linkage maps, and enabling germplasm identification and marker-assisted breeding [24,25]. For instance, using SSR markers, Yan et al. successfully clustered 342 maize accessions and elucidated their pedigree relationships and genetic diversity, which highlighted the critical role of SSRs in deciphering genetic structure and evolutionary relationships [26].

Next-generation sequencing (NGS) technologies have revolutionized large-scale SSR marker development programs. Recent studies have extensively utilized transcriptomic or genomic data to identify SSR markers for plant genetic diversity analysis [27,28]. While the development of transcriptome-derived SSRs remains a commonly used approach, transcriptome-derived SSRs’ distribution patterns differ significantly from genomic SSRs. For example, Yu et al. compared SSR distributions in the genome (14,733 loci) and transcriptome (5411 loci) of *Lycium barbarum*, and found that trinucleotide repeats dominated the genome (66.51%), whereas dinucleotide repeats prevailed in the transcriptome (49.27%). This discrepancy suggests that transcriptome-based approaches may overlook numerous polymorphic loci [29].

With the declining cost of DNA sequencing, high-quality reference genomes and genome resequencing projects have advanced rapidly. As of 2023, genome resequencing had been applied to 187 plant species [23]. Compared to transcriptome- or genome-derived SSR development, whole-genome resequencing (WGR) enables more efficient identification of polymorphic SSR loci. In this study, we obtained 168,459 sequences on single nucleotide polymorphisms and long-fragment insertions from the WGR data of 60 *A. sinensis* accessions, and produced 56,657 SSR sequences (24,430 loci) from 168,459 sequences, with dinucleotide repeats (73.59%) being the most abundant, followed by trinucleotide repeats (16.86%). This pattern is consistent with the transcriptome-based SSR findings in *A. sinensis* [13].

The wild populations of *A. sinensis*, a key economic and ecological species in Hainan Province, have suffered a severe decline due to overexploitation for medicinal and aromatic uses. The challenge in maintaining diversity in its population is further compounded by the ambiguity in the genetic background of the cultivated stocks. To address this, researchers have focused on genetic diversity and population structure analysis [22,30]. In this study, we developed SSR markers using resequencing data and selected 20 loci (*PIC*: 0.196–0.688; mean: 0.507) to analyze 149 accessions. These markers have classified the germplasm into three subpopulations with 95.97% identification accuracy. Our study identified fewer subpopulations (*K* = 3) compared to Xu et al.’s 16-subgroup classification. This divergence likely reflects our intentional sampling bias toward cultivated varieties with demonstrated high agarwood-yielding traits, which may possess reduced genetic diversity due to selective breeding practices [13]. Notably, both studies demonstrated that the clustering patterns of *A. sinensis* show no significant correlation with geographic distribution. This discordance likely reflects extensive anthropogenic translocations [13]. Compared to traditional transcriptome-based methods, WGR provides broader genome coverage and identifies greater numbers of highly polymorphic SSR loci; it enriches the marker resources for molecular ID development. The SSR system established here not only accurately differentiates *A. sinensis* accessions but also enhances genetic diversity analysis, laying a foundation for germplasm conservation, molecular breeding, and genetic research in this species. In subsequent studies, we will continue to conduct research such as multiplex PCR and GWAS based on these 20 SSR markers, with the aim of finding key SSR markers to enhance agarwood production and for application in *A. sinensis* breeding.

## 4. Materials and Methods

### 4.1. DNA Extraction

Fresh leaf samples were collected from 149 *A. sinensis* accessions (Table S6). The samples were homogenized using a Biosample High-throughput Horizontal Mill system (Suzhen Biotechnology, Hangzhou, China). Genomic DNA was extracted using the Plant Genomic DNA Extraction Kit (Tiangen Biotech, Beijing, China). DNA quality was assessed with the help of 1% agarose gel electrophoresis, and purity was measured using an OSE-260 ultra-micro spectrophotometer (Tiangen Biotech, Beijing, China). Samples with clear electrophoretic bands, A260/A280 ratios of 1.8–2.0, and A260/A230 ratios ≥ 2.0 were selected and stored at −80 °C for subsequent use.

### 4.2. SSR Locus Identification and Primer Design

Based on the WGR data of 60 *A. sinensis* accessions, VCF files corresponding to the resequencing of these accessions were used to derive insertion/deletion (InDel) variants with lengths > 10 nt [31]. SSR loci were identified using MISA software (https://webblast.ipk-gatersleben.de/misa/) with the criterion that minimum repeat units of 10, 6, 5, 5, 5, and 5 were used for mono-, di-, tri-, tetra-, penta-, and hexanucleotide motifs, respectively. Compound SSR loci and mononucleotide repeats were excluded. Quality control was performed on the basis of variant quality, minor allele frequency (MAF), and missing data rates. Specifically, loci were retained if they met the following criteria: (1) variant quality score (QUAL) > 40 and genotype quality score (GQ) > 40 so that the reliability of variant calling is ensured; (2) sequencing depth (DP) between 5 and 100, so that low-coverage inaccuracies or high-coverage noises are avoided; and (3) MAF < 0.05 (to prioritize low-frequency variants) or the missing data rate (F_MISSING) ≤ 5%. Primer pairs were designed by extending the sequence by 150 bp upstream and downstream of each SSR locus in the reference genome. The primer parameters were set as follows: minimum, optimal, and maximum melting temperatures (Tm) of 57.0 °C, 60.0 °C, and 61.0 °C, respectively (maximum Tm difference between primers was set to 5 °C); primer lengths of 18–27 bp (optimal: 20 bp); GC content of 20–80%; and PCR product sizes of 100–300 bp.

### 4.3. SSR-Based Genotyping

Primers yielding stable amplification with ≥3 distinct bands were selected for genotyping. PCR reactions were performed in 10 μL volumes containing 5 μL 2× Taq PCR Master Mix (GeneTech, Shanghai, China), 0.5 μL each of forward and reverse primers (10 pmol/μL), 1 μL DNA template (about 20 ng), and 3 μL ddH_2_O. The amplification conditions included initial denaturation at 95 °C for 5 min; 10 cycles of 95 °C for 30 s, 62–52 °C (gradient) for 30 s, and 72 °C for 30 s; 25 cycles of 95 °C for 30 s, 52 °C for 30 s, and 72 °C for 30 s; a final extension at 72 °C for 20 min; and a hold at 4 °C. The reactions were conducted in a Veriti384 PCR Thermal Cycler (Applied Biosystems, Waltham, MA, USA).

To ensure the specificity of fluorescent PCR amplification and the concentration uniformity of the samples for capillary electrophoresis, after completing the fluorescent PCR amplification, 2 μL of the PCR products was subjected to agarose gel electrophoresis (1% concentration). The banding patterns of the PCR products were used to evaluate the amplification specificity of each SSR primer, while the band intensity was used to assess the amplification efficiency. According to the concentration requirements for sample detection, all of the fluorescent PCR products were diluted to obtain uniformly concentrated fluorescent PCR products, which were then prepared for sequencing instrument detection.

The diluted fluorescent PCR products with standardized concentrations were loaded onto the detection plate, and the following detection reagent system was added separately: 1.0 μL of fluorescent PCR product, 0.5 μL of GeneScan™ 500 LIZ (AppliedBiosystem, Waltham, MA, USA), and 8.5 μL Hi-Di™ Formamide (AppliedBiosystem, Waltham, MA, USA). The prepared detection plate containing the samples and reagents was centrifuged and then placed in a Veriti384 PCR Thermal Cycler (Applied Biosystems, Waltham, MA, USA) to run the denaturation program (95 °C, 3 min). After denaturation, the samples were immediately cooled. Following the ABI 3730xL (AppliedBiosystem, Waltham, MA, USA) operating procedure, the corresponding detection file for the plate name was selected, and the SSR sample analysis detection program was executed.

The amplified products were separated with the help of fluorescent capillary electrophoresis and were analyzed using GeneMarker v2.2.0 (SoftGenetics, State College, PA, USA) to determine allele numbers, peak patterns, and genotypes.

### 4.4. SSR Polymorphism and Genetic Diversity Analysis

GenAlex6.5 was used to calculate the genetic parameters for 20 primer pairs, which included the observed alleles (*Na*), effective alleles (*Ne*), Shannon’s information index (*I*), expected heterozygosity (*He*), observed heterozygosity (*Ho*), and fixation index (*F*) [32]. Polymorphism information content (*PIC*) was determined by using Cervus3.0 (http://www.fieldgenetics.com (accessed on 24 August 2024)) [33]. Population structure analysis was performed with the help of Structure2.3.4, with K-values (hypothetical subpopulations) ranging from 1 to 20. Each K was run 20 times with a burn-in period of 10,000 iterations and 100,000 Markov chain monte carlo (MCMC) replicates [34]. The optimal K was determined with the help of the ΔK method and using StructureSelector (https://lmme.ac.cn/StructureSelector/ (accessed on 9 August 2024)) [35]. Genetic structure visualization, molecular variance analysis (AMOVA), and UPGMA clustering, based on *Nei’s* genetic distance, were conducted using Excel, GenAlex6.5, and R4.4.1 (https://www.r-project.org/ (accessed on 5 August 2024)), respectively [32,36]. A binary matrix (0/1) was generated to represent SSR banding patterns for fingerprinting.

## 5. Conclusions

This study developed a large number of SSR loci using resequencing data from 60 *A. sinensis* accessions and designed primers to screen 20 highly efficient and reliable SSR primer pairs. These markers were successfully applied to analyze the genetic diversity of 149 *A. sinensis* accessions and to construct fingerprints, which achieved a discrimination rate of 95.97%. Our findings demonstrate that SSR marker development from resequencing data not only enables more efficient identification of SSR loci but also provides a robust tool for genetic diversity analysis in *A. sinensis* populations.

## Figures and Tables

**Table 1:** SSR locus characteristics.

SSR Locus Characteristics	Quantity
Total number of identified sequences	168,459
SSR sequences	56,657
SSR loci	24,430
Mononucleotide repeats	4747
Dinucleotide repeats	41,695
Trinucleotide repeats	9550
Tetranucleotide repeats	644
Pentanucleotide repeats	13
Hexanucleotide repeats	8

**Table 2:** Characteristics of 20 polymorphic SSR primers.

SSR Primer	SSR Locus ID	Motif	Forward Primer Sequence	Reverse Primer Sequence
AquSSR07	Scaffold_5532_102997041	CA	AAACGGAACGTGCTAATGCT	CCACACGTGATTTCTGGATG
AquSSR10	Scaffold_9996_84841276	ATG	TATTAGTGGGTGAATCGGGC	GGGCAAACGGTATAATCATCA
AquSSR14	Scaffold_15334_39078260	AT	GACATAAGGGGCCATGAGTG	AAGCCTAGCCTTTTTGGTGG
AquSSR17	Scaffold_10796_83936585	AAT	CAAAACCAAATTCACTTGAAAGC	CCACCAGCACAAGTGGTATG
AquSSR18	Scaffold_10796_86795031	ATT	GGAGAGGGTTGAGGTAGGGT	CGGTGTTTGAGATTGTGGAA
AquSSR22	Scaffold_8152_21061737	TG	GGAACTCAATAGGCTGCTGG	CAAATTTTGGGTTGGGTACG
AquSSR27	Scaffold_3585_12180739	TTC	CATTTTACTTTTTGGCGGGA	TGCAACACAAGCAACACAAA
AquSSR28	Scaffold_3585_77017729	AAT	CGAGTGAGGGTTCACCAACT	TGCTCCATAAATGCATGCTC
AquSSR29	Scaffold_10546_3054275	GAA	AACACCTTCATCACCGGAAG	GGGCTTTTGTCATTTTCCCT
AquSSR30	Scaffold_10546_7777087	GA	TTAGCATGGTTTTGTGCTGG	TGCACAACCTCCTCTCTGTG
AquSSR34	Scaffold_10546_70298677	ATAC	ACCATGGACCACAGAGAAGC	AAGGGTATGTGTTGAAGGCG
AquSSR40	Scaffold_10433_55273834	TTA	TCTCCCACGTTTCCAACTTC	TTTGGTCACGAAAAGTGGTG
AquSSR42	Scaffold_10433_59579622	TAT	AACCCTTGTTTGAATGCAGG	CCTAATGGCTGAAAGCCTGA
AquSSR54	Scaffold_10796_47706258	TTA	TGCCCTTTAGACCATGGAAG	AGACCAATAGACCCAAGATGG
AquSSR58	Scaffold_3585_1832452	AAC	CAATGGGGTTTCTACAGGCA	TTGTTGGACATCACAAACGG
AquSSR59	Scaffold_3585_64368533	GCT	AGGGGAGGTGAAGAAAAGGA	CCATAACCATAGCAGCAGCA
AquSSR62	Scaffold_10546_2555776	ATA	TGTGTGGGTAAAATGAAGGCT	TGCCTAAATCTCCTTTGCTTTC
AquSSR71	Scaffold_5532_21554441	AAG	CGCAACCTCATGGGTAACTT	AACCAATCCTCAAACCTCCC
AquSSR89	Scaffold_10546_79030278	TAA	TTTTAATCAGGGGAGGACCC	TCTGCTGACGTGTACGGTTC
AquSSR94	Scaffold_10433_72319168	TAA	CCACTGTTTCTGCAAGCTAGG	GACTTCGTGATCTCAACGGG

Note: For clarity in subsequent analyses, all locus identifiers have been replaced with their corresponding primer IDs (e.g., locus “Scaffold_5532_102997041” is now designated by its primer pair “AquSSR07”).

**Table 3:** Polymorphic characteristics of 20 SSR loci across 149 *Aquilaria sinensis* accessions.

Locus	*N*	*Na*	*Ne*	*I*	*Ho*	*He*	*F*	*PIC*
AquSSR07	149	3	2.014	0.820	0.423	0.503	0.160	0.422
AquSSR10	149	6	3.751	1.403	0.725	0.733	0.012	0.688
AquSSR14	149	6	1.256	0.478	0.195	0.204	0.047	0.196
AquSSR17	149	4	1.410	0.578	0.282	0.291	0.031	0.268
AquSSR18	149	10	2.049	1.168	0.389	0.512	0.240	0.489
AquSSR22	149	4	3.048	1.193	0.181	0.672	0.730	0.608
AquSSR27	149	7	3.599	1.456	0.497	0.722	0.312	0.676
AquSSR28	149	6	1.891	0.963	0.389	0.471	0.174	0.443
AquSSR29	149	12	2.496	1.282	0.671	0.599	−0.120	0.564
AquSSR30	149	7	3.148	1.236	0.564	0.682	0.174	0.617
AquSSR34	148	3	1.344	0.493	0.250	0.256	0.023	0.236
AquSSR40	148	7	3.308	1.457	0.615	0.698	0.119	0.660
AquSSR42	148	5	2.904	1.192	0.581	0.656	0.114	0.595
AquSSR54	149	9	2.680	1.221	0.631	0.627	−0.006	0.564
AquSSR58	149	5	2.074	0.864	0.477	0.518	0.080	0.434
AquSSR59	148	5	3.706	1.365	0.716	0.730	0.019	0.680
AquSSR62	149	6	2.627	1.098	0.550	0.619	0.112	0.554
AquSSR71	148	5	1.939	0.832	0.405	0.484	0.163	0.410
AquSSR89	145	3	1.964	0.781	0.428	0.491	0.129	0.402
AquSSR94	147	8	3.009	1.389	0.932	0.668	−0.396	0.627
Mean	148.45	6.050	2.511	1.063	0.495	0.557	0.106	0.507

Note: *Na*, number of alleles; *Ne*, effective number of alleles; *I*, Shannon’s information index; *Ho*, observed heterozygosity; *He*, expected heterozygosity; *F*, fixation index = (*He* − *Ho*)/*He*; *PIC*, polymorphic information content.

**Table 4:** Genetic diversity indices of three *A. sinensis* clusters.

Population	*N*	*Na*	*Ne*	*I*	*Ho*	*He*	*F*
I	70.750	4.600	2.280	0.934	0.484	0.505	0.050
II	29.750	4.800	2.816	1.141	0.561	0.603	0.063
III	47.950	3.850	2.141	0.851	0.471	0.470	−0.002
Mean	49.483	4.417	2.412	0.975	0.505	0.526	0.037

Note: *Na*, number of alleles; *Ne*, effective number of alleles; *I*, Shannon’s information index; *Ho*, observed heterozygosity; *He*, expected heterozygosity; *F*, fixation index = (*He* − *Ho*)/*He*.

**Table 5:** Analysis of molecular variance (AMOVA) for *A. sinensis* clusters.

Source of Variation	Degrees of Freedom (df)	Sum of Squares (SS)	Mean Squares (MS)	Estimated Variance	Percentage of Variation (%)
Among Populations	2	130.156	65.078	0.636	11%
Within Populations	146	804.267	5.509	0.290	5%
Among Individuals	149	734.500	4.930	4.930	84%
Total	297	1668.923		5.855	100%

**Table 6:** Pairwise *Fst* values among three *A. sinensis* clusters.

Population	Ⅰ	Ⅱ	Ⅲ
I	0.000		
II	0.056	0.000	
III	0.051	0.085	0.000

## Data Availability

The original contributions presented in this study are included in the article/Supplementary Material. Further inquiries can be directed to the corresponding authors.

## Supplementary Materials

The following supporting information can be downloaded at: https://www.mdpi.com/article/10.3390/plants14091323/s1, Figure S1. Fingerprint profiles of 149 A. sinensis accessions based on 20 SSR Loci. In the fingerprint map, each row horizontally represents an accession, while each color vertically indicates the amplification status of an SSR locus across different accessions. Table S1. Information of SSR sequencing. Table S2. Frequency statistics of SSR genotypes. Table S3. 93 pairs of SSR primer primary screening I results. Table S4. 23 pairs of SSR primer primary screening II results. Table S5. Three genetic clusters identified by STRUCTURE analysis. Table S6. Information about 149 *A. sinensis* accessions.
