# Supplementary material for: Development and Application of SSR Markers for Aquilaria sinensis on the Basis of Whole-Genome Resequencing Data
Source: Plants (Basel). 2025 Apr 27;14(9):1323. doi: 10.3390/plants14091323 (PMC12073285; doi:10.3390/plants14091323)
Supplement: Supplementary file 1 [file plants-14-01323-s001.zip › plants-3565628-supplementary.pdf]

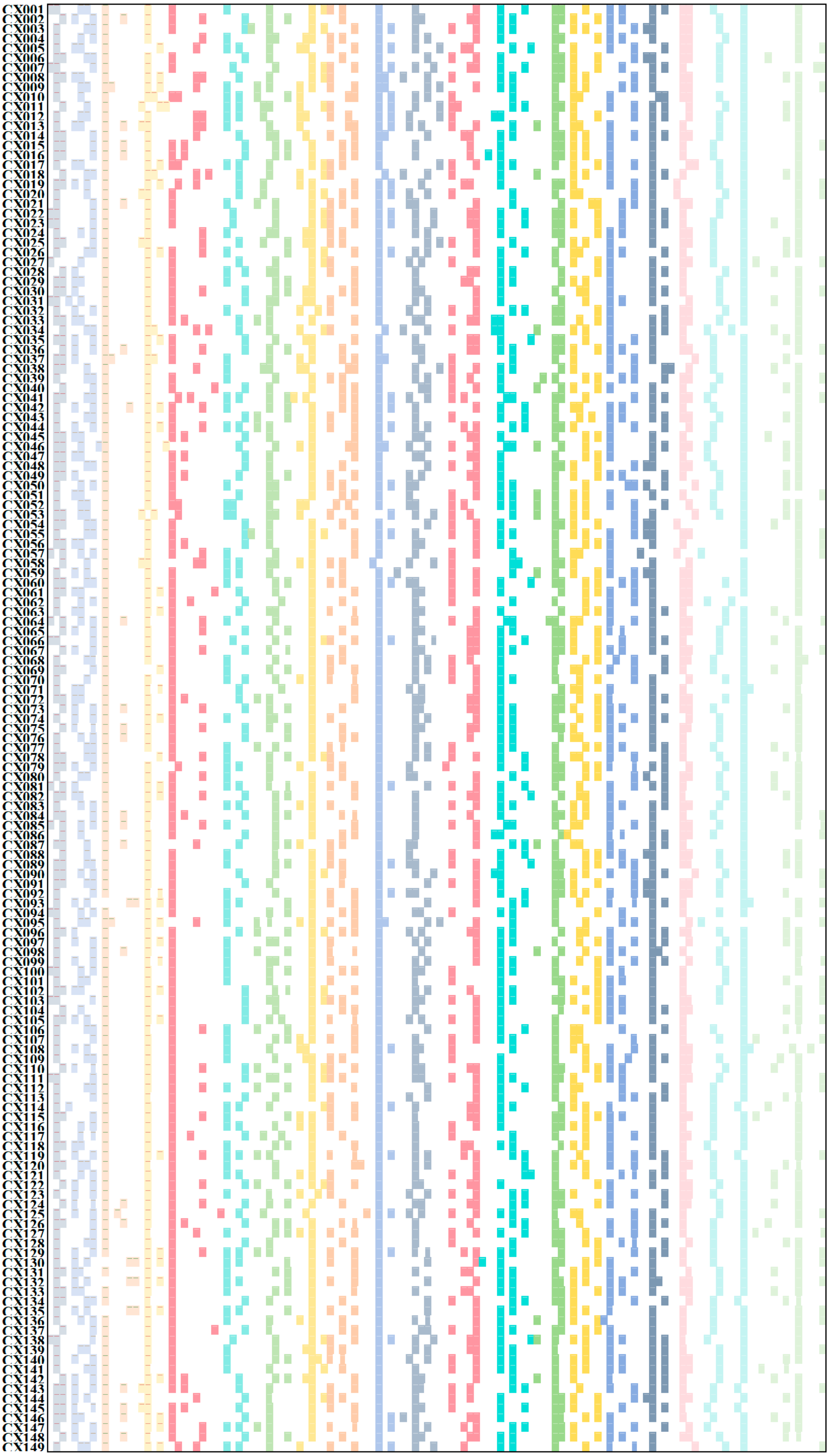

Figure S1. Fingerprint profiles of 149 *A. sinensis* accessions based on 20 SSR Loci. In the fingerprint map, each row horizontally represents an accession, while each color vertically indicates the amplification status of an SSR locus across different accessions.

Table S1. Information of SSR sequencing.

Type	Motif	Number	Percentage
mononucleotide repeats	A/T	3013	63.47%
	C/G	1734	36.53%
	Total	4747	100.00%
dinucleotide repeats	AT/AT	15194	36.44%
	TA/TA	12840	30.80%
	TG/CA	3879	9.30%
	AC/GT	3544	8.50%
	AG/CT	3148	7.55%
	TC/GA	3065	7.35%
	GC/GC	14	0.03%
	CG/CG	11	0.03%
	Total	41695	100.00%
trinucleotide repeats	TAT/ATA	3007	31.49%
	AAT/ATT	2789	29.20%
	TTA/TAA	2749	28.79%
	TTC/GAA	207	2.17%
	AAG/CTT	187	1.96%
	AGA/TCT	134	1.40%
	TTG/CAA	86	0.90%
	AAC/GTT	67	0.70%
	ATC/GAT	65	0.68%
	TAG/CTA	57	0.60%
	ATG/CAT	46	0.48%
	TAC/GTA	46	0.48%
	TCA/TGA	19	0.20%
	ACT/AGT	19	0.20%
	ACA/TGT	12	0.13%
	AGG/CCT	9	0.09%
	GGA/TCC	8	0.08%
	GAG/CTC	7	0.07%
	CCA/TGG	7	0.07%
	GCT/AGC	6	0.06%
	GCC/GGC	5	0.05%
	ACC/GGT	5	0.05%
	CAC/GTG	4	0.04%
	CAG/CTG	4	0.04%
	GCA/TGC	2	0.02%
	GAC/GTC	1	0.01%
	CGG/CCG	1	0.01%
	TCG/CGA	1	0.01%
	Total	9550	100.00%
Tetranucleotide repeats	TATG/CATA	228	35.40%
	ATAC/GTAT	135	20.96%
	ATGT/ACAT	72	11.18%
	TACA/TGTA	63	9.78%
	AATA/TATT	31	4.81%
	AAAT/ATTT	18	2.80%
	TTTA/TAAA	16	2.48%
	TTAT/ATAA	12	1.86%
	AAAG/CTTT	11	1.71%
	TTTC/GAAA	10	1.55%
	TATC/GATA	8	1.24%
	TTGT/ACAA	4	0.62%
	AAAC/GTTT	3	0.47%
	TGGA/TCCA	3	0.47%

	TTAA/TTAA	3	0.47%
	AGAA/TTCT	3	0.47%
	AGTT/AACT	2	0.31%
	ATCT/AGAT	2	0.31%
	CCCT/AGGG	2	0.31%
	CTAT/ATAG	2	0.31%
	GACA/TGTC	2	0.31%
	TCTT/AAGA	2	0.31%
	ACAG/CTGT	1	0.16%
	ACCC/GGGT	1	0.16%
	ATTA/TAAT	1	0.16%
	ATTG/CAAT	1	0.16%
	CAAA/TTTG	1	0.16%
	GAGG/CCTC	1	0.16%
	GATG/CATC	1	0.16%
	GCAG/CTGC	1	0.16%
	TAGA/TCTA	1	0.16%
	TAGT/ACTA	1	0.16%
	TCAA/TTGA	1	0.16%
	TTAG/CTAA	1	0.16%
	Total	644	100.00%
Pentanucleotide repeats	AATAT/ATATT	4	30.77%
	TTTTA/TAAAA	3	23.08%
	AATTT/AAATT	1	7.69%
	AGCCT/AGGCT	1	7.69%
	GAAGG/CCTTC	1	7.69%
	TATCT/AGATA	1	7.69%
	TGTTT/AAACT	1	7.69%
	TTGTG/CACAA	1	7.69%
	Total	13	100.00%
Hexanucleotide repeats	ACGAAC/GTTCGT	2	25.00%
	CCTCTC/GAGAGG	2	25.00%
	AATTCA/TGAATT	1	12.50%
	GTGCGT/ACGCAC	1	12.50%
	TGTTCG/CGAACA	1	12.50%
	TTTTAT/ATAAAA	1	12.50%
	Total	8	100.00%

Table S2. Frequency statistics of SSR genotypes.

SSR ID	Motif	Most common gene frequency	Second most common gene frequency	Third most common gene frequency
Scaffold_5532_671225	TA	0.62	0.15	0.15
Scaffold_5532_5578186	TAT	0.77	0.12	0.08
Scaffold_5532_7333135	TG	0.77	0.15	0.07
Scaffold_5532_8308141	GCT	0.22	0.17	0.15
Scaffold_5532_9628866	ATT	0.40	0.38	0.08
Scaffold_5532_11022017	AC	0.53	0.40	0.03
Scaffold_5532_28797113	TTC	0.62	0.23	0.15
Scaffold_5532_63506385	TTA	0.43	0.35	0.10
Scaffold_5532_75893305	ATAC	0.57	0.22	0.08
Scaffold_5532_85031602	TG	0.55	0.37	0.07
Scaffold_5532_87027194	TAT	0.38	0.32	0.13
Scaffold_5532_88552548	ATT	0.83	0.17	0.00
Scaffold_5532_90050954	AAT	0.75	0.12	0.10
Scaffold_5532_91642329	AAT	0.47	0.40	0.12
Scaffold_5532_91909625	CAT	0.68	0.18	0.07
Scaffold_5532_93060318	AC	0.62	0.33	0.05
Scaffold_5532_93697752	AGT	0.58	0.23	0.08
Scaffold_5532_93852064	TAT	0.68	0.13	0.13
Scaffold_5532_94724321	AT	0.77	0.15	0.02
Scaffold_5532_94737050	CA	0.40	0.30	0.12
Scaffold_5532_95628583	ACAT	0.70	0.22	0.03
Scaffold_5532_96348009	TA	0.63	0.18	0.03
Scaffold_5532_99422980	TC	0.75	0.13	0.05
Scaffold_5532_101473823	TGG	0.87	0.12	0.02
Scaffold_5532_102010635	CT	0.47	0.38	0.12
Scaffold_5532_102997041	CA	0.43	0.32	0.13
Scaffold_5532_103380551	CTT	0.55	0.40	0.03
Scaffold_5532_103658454	ACAT	0.33	0.32	0.12
Scaffold_5532_103985286	AT	0.50	0.47	0.02
Scaffold_5532_104643030	AT	0.43	0.32	0.15
Scaffold_5532_105546731	TAT	0.55	0.35	0.07
Scaffold_5532_107295359	AAT	0.33	0.22	0.17

Scaffold_5532_109254228	CTT	0.15	0.15	0.13
Scaffold_5532_109866451	GAA	0.57	0.20	0.17
Scaffold_9996_181604	AG	0.65	0.23	0.03
Scaffold_9996_1399494	TA	0.58	0.27	0.08
Scaffold_9996_3581509	GT	0.63	0.28	0.07
Scaffold_9996_3957853	TAT	0.58	0.35	0.05
Scaffold_9996_7815913	CA	0.75	0.23	0.02
Scaffold_9996_72481705	AGT	0.78	0.17	0.03
Scaffold_9996_76647981	AAG	0.40	0.35	0.05
Scaffold_9996_83100715	AG	0.78	0.13	0.02
Scaffold_9996_84841276	ATG	0.27	0.20	0.15
Scaffold_9996_86197166	TG	0.68	0.27	0.03
Scaffold_9996_89147194	TA	0.62	0.33	0.02
Scaffold_9996_89482031	TA	0.80	0.18	0.02
Scaffold_9996_89522506	ATT	0.53	0.35	0.03
Scaffold_9996_89808358	CA	0.63	0.25	0.07
Scaffold_9996_92947270	GAA	0.60	0.17	0.12
Scaffold_9996_93413664	AC	0.60	0.25	0.07
Scaffold_15334_980867	AAT	0.45	0.35	0.13
Scaffold_15334_3270945	AT	0.42	0.27	0.17
Scaffold_15334_3527407	TAA	0.47	0.25	0.23
Scaffold_15334_8333814	AAT	0.70	0.28	0.02
Scaffold_15334_11611799	AAT	0.32	0.20	0.17
Scaffold_15334_11613357	CA	0.32	0.18	0.17
Scaffold_15334_16697539	CTG	0.48	0.35	0.17
Scaffold_15334_21047423	TTC	0.65	0.18	0.05
Scaffold_15334_22363251	ATT	0.77	0.20	0.02
Scaffold_15334_39078260	AT	0.70	0.17	0.10
Scaffold_15334_75576382	ATT	0.80	0.13	0.03
Scaffold_10796_20188058	TTTA	0.77	0.22	0.02
Scaffold_10796_47706258	TTA	0.37	0.23	0.15
Scaffold_10796_69322944	AC	0.42	0.42	0.08
Scaffold_10796_77149115	GAA	0.48	0.37	0.15
Scaffold_10796_78322094	ATC	0.75	0.17	0.07
Scaffold_10796_78388626	CA	0.82	0.15	0.00

Scaffold_10796_79176749	ATT	0.53	0.28	0.12
Scaffold_10796_82799245	AGC	0.73	0.12	0.10
Scaffold_10796_83936585	AAT	0.68	0.23	0.05
Scaffold_10796_84391461	AC	0.48	0.33	0.15
Scaffold_10796_85215419	TA	0.32	0.20	0.17
Scaffold_10796_85295842	ATA	0.53	0.15	0.15
Scaffold_10796_86065226	TAT	0.40	0.37	0.15
Scaffold_10796_86795031	ATT	0.77	0.22	0.00
Scaffold_10796_87400131	ATA	0.33	0.28	0.13
Scaffold_10796_87780628	AT	0.57	0.37	0.07
Scaffold_8152_150911	AC	0.38	0.30	0.18
Scaffold_8152_2082387	AT	0.47	0.38	0.10
Scaffold_8152_5549609	AAT	0.42	0.30	0.23
Scaffold_8152_6096913	CTT	0.73	0.20	0.03
Scaffold_8152_7055688	TG	0.43	0.32	0.23
Scaffold_8152_8716527	TA	0.72	0.17	0.08
Scaffold_8152_12072172	AT	0.43	0.33	0.22
Scaffold_8152_16679434	TTG	0.40	0.30	0.28
Scaffold_8152_16791079	AC	0.57	0.27	0.08
Scaffold_8152_19709001	TTA	0.87	0.10	0.02
Scaffold_8152_21061737	GT	0.72	0.23	0.03
Scaffold_8152_71459705	TG	0.65	0.33	0.02
Scaffold_8152_74021183	AAG	0.50	0.13	0.12
Scaffold_8152_74824704	TAA	0.73	0.10	0.07
Scaffold_8152_80030444	AC	0.77	0.13	0.05
Scaffold_8152_81824977	AT	0.45	0.25	0.22
Scaffold_8152_82301387	GAG	0.37	0.27	0.25
Scaffold_8152_82626912	ATG	0.65	0.20	0.08
Scaffold_8152_83234068	TAA	0.78	0.22	0.00
Scaffold_8152_84445404	TA	0.72	0.15	0.07
Scaffold_8152_85216677	TG	0.60	0.33	0.03
Scaffold_8152_86919786	AT	0.87	0.08	0.03
Scaffold_3585_560299	AC	0.78	0.15	0.02
Scaffold_3585_1824254	TTG	0.42	0.20	0.20
Scaffold_3585_1832452	AAC	0.40	0.37	0.13

Scaffold_3585_6042398	AT	0.68	0.17	0.05
Scaffold_3585_8642443	AAG	0.82	0.18	0.00
Scaffold_3585_12180739	TCT	0.67	0.30	0.03
Scaffold_3585_56548933	CA	0.28	0.20	0.18
Scaffold_3585_64368533	GCT	0.22	0.17	0.13
Scaffold_3585_67783805	TA	0.70	0.17	0.10
Scaffold_3585_71854425	AAT	0.57	0.30	0.05
Scaffold_3585_72943661	GT	0.65	0.27	0.05
Scaffold_3585_73997513	TTA	0.65	0.18	0.07
Scaffold_3585_75179068	AC	0.52	0.23	0.08
Scaffold_3585_77017729	AAT	0.57	0.18	0.12
Scaffold_3585_80954147	TCA	0.40	0.35	0.10
Scaffold_10546_1511198	TA	0.22	0.18	0.17
Scaffold_10546_2555776	ATA	0.37	0.23	0.20
Scaffold_10546_3054275	GAA	0.47	0.27	0.18
Scaffold_10546_7777087	GA	0.60	0.23	0.17
Scaffold_10546_13990289	TACA	0.48	0.20	0.17
Scaffold_10546_14656175	AT	0.38	0.35	0.10
Scaffold_10546_17418438	TC	0.60	0.25	0.10
Scaffold_10546_24531616	TTC	0.53	0.15	0.13
Scaffold_10546_25568559	TA	0.43	0.32	0.13
Scaffold_10546_26276781	TC	0.78	0.20	0.02
Scaffold_10546_29027110	AC	0.22	0.22	0.20
Scaffold_10546_32442278	TAA	0.70	0.25	0.05
Scaffold_10546_59241145	CA	0.77	0.22	0.00
Scaffold_10546_62233195	AT	0.58	0.33	0.07
Scaffold_10546_70298677	ATAC	0.85	0.12	0.03
Scaffold_10546_75226386	TCT	0.72	0.17	0.08
Scaffold_10546_76593389	TTA	0.32	0.27	0.15
Scaffold_10433_2007303	TC	0.38	0.32	0.13
Scaffold_10433_4577082	ATG	0.38	0.25	0.13
Scaffold_10433_6501504	GGC	0.57	0.30	0.05
Scaffold_10433_9037643	TC	0.75	0.22	0.02
Scaffold_10433_9138406	ATT	0.78	0.20	0.02
Scaffold_10433_9932354	AAT	0.73	0.17	0.03

Scaffold_10433_29701796	TG	0.63	0.32	0.05
Scaffold_10433_49834618	TTC	0.47	0.25	0.15
Scaffold_10433_55273834	TTA	0.30	0.25	0.20
Scaffold_10433_56664634	TG	0.63	0.25	0.05
Scaffold_10433_58153581	CTT	0.37	0.35	0.10
Scaffold_10433_59541503	ATAC	0.50	0.17	0.10
Scaffold_10433_59579622	TAT	0.53	0.27	0.08
Scaffold_10433_62854018	TAT	0.53	0.23	0.13
Scaffold_10433_68197891	ATT	0.80	0.13	0.03
Scaffold_10433_71653783	CTT	0.78	0.12	0.07
Scaffold_10433_72351300	TC	0.57	0.28	0.13
Scaffold_10433_75958727	AT	0.58	0.38	0.02
Scaffold_10433_76788146	ACAT	0.70	0.12	0.07
Scaffold_8660_3413	GCT	0.68	0.27	0.03
Scaffold_13652_665	TTA	0.68	0.23	0.03

Table S3. 93 pairs of SSR primer primary screening I results.

SSR Primer	Motif	Length of target fragment（bp）	Capillary electrophoresis detection
AquSSR01	GC	112	Good
AquSSR02	ATT	204	Good
AquSSR03	ATA	226	Good
AquSSR04	AC	110	Good
AquSSR05	TAT	109	Good
AquSSR06	TC	133	Good
AquSSR07	CA	149	Good
AquSSR08	AC	110	Good
AquSSR09	AG	201	Good
AquSSR10	ATG	123	Good
AquSSR11	AC	278	Good
AquSSR12	CA	147	Good
AquSSR13	TGC	119	Good
AquSSR14	AT	182	Good
AquSSR15	ATT	214	Stutter peaks
AquSSR16	CA	140	Stutter peaks
AquSSR17	AAT	200	Good
AquSSR18	ATT	154	Good
AquSSR19	TA	110	Good
AquSSR20	TTG	152	Good
AquSSR21	AC	254	Good
AquSSR22	TG	197	Good
AquSSR23	AT	161	Good
AquSSR24	AAT	105	Good
AquSSR25	TA	237	Good
AquSSR26	AAG	202	Good
AquSSR27	TTC	121	Good
AquSSR28	AAT	217	Good
AquSSR29	GAA	123	Good
AquSSR30	GA	192	Good
AquSSR31	TC	265	Good
AquSSR32	TAA	149	Good
AquSSR33	CA	124	Good

AquSSR34	ATAC	202	Good
AquSSR35	TCT	129	Good
AquSSR36	GGC	218	Good
AquSSR37	TC	103	Good
AquSSR38	TAT	159	Stutter peaks
AquSSR39	TG	128	Stutter peaks
AquSSR40	TTA	138	Good
AquSSR41	CTT	127	Good
AquSSR42	TAT	163	Good
AquSSR43	ATT	173	Stutter peaks
AquSSR44	CTT	137	Good
AquSSR45	ATAC	151	Good
AquSSR46	GCT	186	Good
AquSSR47	GCT	135	Good
AquSSR48	TTC	249	Good
AquSSR49	TTA	239	Good
AquSSR50	GAA	172	Good
AquSSR51	AT	170	Good
AquSSR52	TAA	148	Good
AquSSR53	AAT	178	Good
AquSSR54	TTA	237	Good
AquSSR55	GAA	217	Good
AquSSR56	AGC	150	Good
AquSSR57	TTG	167	Stutter peaks
AquSSR58	AAC	178	Good
AquSSR59	GCT	177	Good
AquSSR61	TA	145	Good
AquSSR62	ATA	222	Good, with additional A peaks
AquSSR63	TACA	196	Good
AquSSR64	TTC	141	Good, with additional A peaks
AquSSR65	ATG	110	Good
AquSSR66	TAT	223	Good
AquSSR67	AG	182	Good
AquSSR68	AC	128	Good
AquSSR69	TTC	179	Good

AquSSR70	AGA	114	Good
AquSSR71	AAG	253	Good
AquSSR72	AT	169	Good
AquSSR73	AT	132	Good
AquSSR74	TAT	159	Good
AquSSR75	AC	201	Good, with additional A peaks
AquSSR76	AAC	201	Good, with additional A peaks
AquSSR77	GA	138	Good
AquSSR78	AG	137	Good
AquSSR79	ATT	125	Good
AquSSR80	GA	186	Stutter peaks
AquSSR81	AAT	153	Good
AquSSR82	AAT	209	Good
AquSSR83	TC	266	Good
AquSSR84	AAG	145	Good
AquSSR85	TTC	236	Good
AquSSR86	TG	128	Good
AquSSR87	AAT	227	Good
AquSSR88	ATT	202	Good
AquSSR89	TAA	116	Good
AquSSR90	TA	104	Good
AquSSR91	GAA	164	Good
AquSSR92	AT	212	Good
AquSSR93	TA	120	Good
AquSSR94	TAA	120	Good

Table S4. 23 pairs of SSR primer primary screening II results.

SSR Primer	Motif	Length of target fragment（bp）	Na	PIC	Capillary electrophoresis detection
AquSSR07	CA	149	3	0.497	Success
AquSSR10	ATG	123	5	0.755	Success
AquSSR14	AT	182	4	0.388	Success
AquSSR17	AAT	200	4	0.388	Success
AquSSR18	ATT	154	6	0.613	Success
AquSSR22	TG	197	3	0.515	Success
AquSSR27	TTC	121	5	0.641	Success
AquSSR28	AAT	217	4	0.56	Success
AquSSR29	GAA	123	3	0.354	Success
AquSSR30	GA	192	3	0.555	Success
AquSSR34	ATAC	202	3	0.371	Success
AquSSR40	TTA	138	5	0.587	Success
AquSSR42	TAT	163	3	0.426	Success
AquSSR54	TTA	237	3	0.497	Success-Good
AquSSR58	AAC	178	3	0.468	Success-Good
AquSSR59	GCT	177	4	0.677	Success-Good
AquSSR62	ATA	222	3	0.426	Success-Good, with additional A peaks
AquSSR64	TTC	141	3	0.295	Success-Good, with additional A peaks
AquSSR71	AAG	253	2	0.305	Success
AquSSR73	AT	132	1	0	Success
AquSSR86	TG	128	1	0	Success
AquSSR89	TAA	116	2	0.359	Success
AquSSR94	TAA	120	2	0.11	Success

Table S5. Three genetic clusters identified by STRUCTURE analysis.

Cluster	Accession
Cluster I	CX001, CX003, CX004, CX006, CX007, CX021, CX022, CX023, CX024, CX026, CX027, CX031, CX032, CX039, CX042, CX043, CX044, CX048, CX050, CX054, CX055, CX057, CX059, CX060, CX064, CX066, CX068, CX069, CX070, CX074, CX077, CX078, CX080, CX081, CX083, CX085, CX088, CX089, CX091, CX092, CX093, CX094, CX097, CX099, CX100, CX101, CX103, CX106, CX107, CX109, CX111, CX112, CX113, CX115, CX116, CX119, CX120, CX121, CX123, CX127, CX128, CX129, CX130, CX132, CX134, CX135, CX136, CX138, CX139, CX140, CX141
Cluster II	CX005, CX008, CX009, CX010, CX011, CX012, CX013, CX014, CX017, CX018, CX019, CX020, CX025, CX034, CX037, CX038, CX041, CX046, CX052, CX053, CX058, CX079, CX087, CX090, CX095, CX108, CX125, CX144, CX147, CX149
Cluster III	CX002, CX015, CX016, CX028, CX029, CX030, CX033, CX035, CX036, CX040, CX045, CX047, CX049, CX051, CX056, CX061, CX062, CX063, CX065, CX067, CX071, CX072, CX073, CX075, CX076, CX082, CX084, CX086, CX096, CX098, CX102, CX104, CX105, CX110, CX114, CX117, CX118, CX122, CX124, CX126, CX131, CX133, CX137, CX142, CX143, CX145, CX146, CX148

Table S6. Information about 149 *A. sinensis* accessions.

ID	Collection Location	Type
CX001	Xiadong Village, Dianbai, Guangdong	Cultivated variety
CX002	Shadong, Guanzhu, Dianbai, Guangdong	Cultivated variety
CX003	Wushi, Qiongzhong, Hainan	Cultivated variety
CX004	Wushi, Qiongzhong, Hainan	Cultivated variety
CX005	Tunchang, Hainan	Wild single plant
CX006	Ding'an, Hainan	Cultivated variety
CX007	Ding'an, Hainan	Cultivated variety
CX008	-	Cultivated variety
CX009	Wuzhishan, Hainan	Cultivated variety
CX010	Yunlong, Haikou, Hainan	Cultivated variety
CX011	Yunlong, Haikou, Hainan	Wild variety
CX012	Tunchang, Hainan	Wild variety
CX013	Wenchang, Hainan	Wild variety
CX014	Limushan, Qiongzhong, Hainan	Wild variety
CX015	Wushi, Qiongzhong, Hainan	Cultivated variety
CX016	Wushi, Qiongzhong, Hainan	Cultivated variety
CX017	Xinpo, Haikou, Hainan	Wild variety
CX018	Xinpo, Haikou, Hainan	Wild variety
CX019	Xinpo, Haikou, Hainan	Wild variety
CX020	Xinpo, Haikou, Hainan	Wild variety
CX021	Beitou Village, Dianbai, Guangdong	Cultivated variety
CX022	Ding'an, Hainan	Cultivated variety
CX023	Ding'an, Hainan	Cultivated variety
CX024	Wushi, Qiongzhong, Hainan	Cultivated variety
CX025	Dongdong, Qiongzhong, Hainan	Wild variety
CX026	Wushi, Qiongzhong, Hainan	Cultivated variety
CX027	Wushi, Qiongzhong, Hainan	Cultivated variety
CX028	Wushi, Qiongzhong, Hainan	Cultivated variety
CX029	Wushi, Qiongzhong, Hainan	Cultivated variety
CX030	Wushi, Qiongzhong, Hainan	Cultivated variety
CX031	Shadong, Guanzhu, Dianbai, Guangdong	Cultivated variety
CX032	Wushi, Qiongzhong, Hainan	Cultivated variety
CX033	Wushi, Qiongzhong, Hainan	Cultivated variety
CX034	Wushi, Qiongzhong, Hainan	Cultivated variety
CX035	Da'an, Qiongzhong, Hainan	Cultivated variety
CX036	Wushi, Qiongzhong, Hainan	Cultivated variety
CX037	Chengmai, Hainan	Cultivated variety
CX038	Tunchang, Hainan	Cultivated variety
CX039	Wushi, Qiongzhong, Hainan	Cultivated variety
CX040	Wushi, Qiongzhong, Hainan	Cultivated variety
CX041	Wuzhishan, Hainan	Cultivated variety
CX042	Tangliaodi, Guanzhu, Dianbai, Guangdong	Cultivated variety

CX043	Tangliaodi, Guanzhu, Dianbai, Guangdong	Cultivated variety
CX044	Tangliaodi, Guanzhu, Dianbai, Guangdong	Cultivated variety
CX045	Tangliaodi, Guanzhu, Dianbai, Guangdong	Cultivated variety
CX046	Lingao, Hainan	Cultivated variety
CX047	Lingao, Hainan	Wild variety
CX048	Wenchang, Hainan	Cultivated variety
CX049	Tangliaodi, Guanzhu, Guangdong	Cultivated variety
CX050	Wuzhishan, Hainan	Cultivated variety
CX051	Lingao, Hainan	Cultivated variety
CX052	Dongdong, Qiongzhong, Hainan	Wild variety
CX053	Yunlong, Haikou, Hainan	Cultivated variety
CX054	Ding'an, Hainan	Cultivated variety
CX055	Wushi, Qiongzhong, Hainan	Cultivated variety
CX056	Lingao, Hainan	Cultivated variety
CX057	Ding'an, Hainan	Cultivated variety
CX058	Tunchang, Hainan	Cultivated variety
CX059	Lingao, Hainan	Wild variety
CX060	Tangliaodi, Guanzhu, Dianbai, Guangdong	Cultivated variety
CX061	Tangliaodi, Guanzhu, Dianbai, Guangdong	Cultivated variety
CX062	Team 14, Shuguang Farm, Dianbai, Guangdong	Cultivated variety
CX063	Tangliaodi, Guanzhu, Dianbai, Guangdong	Cultivated variety
CX064	Wangfu, Dianbai, Guangdong	Cultivated variety
CX065	Heping, Guanzhu, Dianbai, Guangdong	Cultivated variety
CX066	Tangliaodi, Guanzhu, Dianbai, Guangdong	Cultivated variety
CX067	Yuntan, Gaozhou, Guangdong	Cultivated variety
CX068	Tangliaodi, Guanzhu, Dianbai, Guangdong	Cultivated variety
CX069	Shadong, Guanzhu, Dianbai, Guangdong	Cultivated variety
CX070	Shadong, Guanzhu, Dianbai, Guangdong	Cultivated variety
CX071	Shadong, Guanzhu, Dianbai, Guangdong	Cultivated variety
CX072	Shadong, Guanzhu, Dianbai, Guangdong	Cultivated variety
CX073	Shadong, Guanzhu, Dianbai, Guangdong	Cultivated variety
CX074	Shadong, Guanzhu, Dianbai, Guangdong	Cultivated variety
CX075	Shadong, Guanzhu, Dianbai, Guangdong	Cultivated variety
CX076	Shadong, Guanzhu, Dianbai, Guangdong	Cultivated variety
CX077	Shadong, Guanzhu, Dianbai, Guangdong	Cultivated variety
CX078	Shadong, Guanzhu, Dianbai, Guangdong	Cultivated variety
CX079	Shenwu, Guanzhu, Dianbai, Guangdong	Cultivated variety
CX080	Zhaojing Village, Guanzhu, Dianbai, Guangdong	Cultivated variety
CX081	Shadong, Guanzhu, Dianbai, Guangdong	Cultivated variety
CX082	Shadong, Guanzhu, Dianbai, Guangdong	Cultivated variety
CX083	Shadong, Guanzhu, Dianbai, Guangdong	Cultivated variety
CX084	Shadong, Guanzhu, Dianbai, Guangdong	Cultivated variety
CX085	Shadong, Guanzhu, Dianbai, Guangdong	Cultivated variety
CX086	Shadong, Guanzhu, Dianbai, Guangdong	Cultivated variety

CX087	Shadong, Guanzhu, Dianbai, Guangdong	Cultivated variety
CX088	Hedong, Guanzhu, Dianbai, Guangdong	Cultivated variety
CX089	Shuijidong, Dianbai, Guangdong	Cultivated variety
CX090	Shuijidong, Dianbai, Guangdong	Cultivated variety
CX091	Lianqun, Mata, Dianbai, Guangdong	Cultivated variety
CX092	Shadong, Guanzhu, Dianbai, Guangdong	Cultivated variety
CX093	Dashuipo, Guanzhu, Dianbai, Guangdong	Cultivated variety
CX094	Shadong, Guanzhu, Dianbai, Guangdong	Cultivated variety
CX095	Shadong, Guanzhu, Dianbai, Guangdong	Cultivated variety
CX096	Juntunpo, Hedong, Dianbai, Guangdong	Cultivated variety
CX097	Juntunpo, Hedong, Dianbai, Guangdong	Cultivated variety
CX098	Juntunpo, Hedong, Dianbai, Guangdong	Cultivated variety
CX099	Shuijidong, Guanzhu, Dianbai, Guangdong	Cultivated variety
CX100	Shuijidong, Guanzhu, Dianbai, Guangdong	Cultivated variety
CX101	Shuijidong, Guanzhu, Dianbai, Guangdong	Cultivated variety
CX102	Shadong, Guanzhu, Dianbai, Guangdong	Cultivated variety
CX103	Shadong, Guanzhu, Dianbai, Guangdong	Cultivated variety
CX104	Shadong, Guanzhu, Dianbai, Guangdong	Cultivated variety
CX105	Shadong, Guanzhu, Dianbai, Guangdong	Cultivated variety
CX106	Shenwu, Shadong, Guanzhu, Dianbai, Guangdong	Cultivated variety
CX107	Shenwu, Shadong, Guanzhu, Dianbai, Guangdong	Cultivated variety
CX108	Shenwu, Shadong, Guanzhu, Dianbai, Guangdong	Cultivated variety
CX109	Shenwu, Shadong, Guanzhu, Dianbai, Guangdong	Cultivated variety
CX110	Shenwu, Shadong, Guanzhu, Dianbai, Guangdong	Cultivated variety
CX111	Shenwu, Shadong, Guanzhu, Dianbai, Guangdong	Cultivated variety
CX112	Shenwu, Shadong, Guanzhu, Dianbai, Guangdong	Cultivated variety
CX113	Shenwu, Shadong, Guanzhu, Dianbai, Guangdong	Cultivated variety
CX114	Shenwu, Shadong, Guanzhu, Dianbai, Guangdong	Cultivated variety
CX115	Wangfu, Dianbai, Guangdong	Cultivated variety
CX116	Shadong, Guanzhu, Dianbai, Guangdong	Cultivated variety
CX117	Shadong, Guanzhu, Dianbai, Guangdong	Cultivated variety
CX118	Shenwu, Shadong, Guanzhu, Dianbai, Guangdong	Cultivated variety
CX119	Shenwu, Shadong, Guanzhu, Dianbai, Guangdong	Cultivated variety
CX120	Shenwu, Shadong, Guanzhu, Dianbai, Guangdong	Cultivated variety
CX121	Shenwu, Shadong, Guanzhu, Dianbai, Guangdong	Cultivated variety
CX122	Shenwu, Shadong, Guanzhu, Dianbai, Guangdong	Cultivated variety
CX123	Shenwu, Shadong, Guanzhu, Dianbai, Guangdong	Cultivated variety
CX124	Pingshan, Guanzhu, Dianbai, Guangdong	Cultivated variety
CX125	Dashuipo, Guanzhu, Dianbai, Guangdong	Cultivated variety
CX126	Fozilou, Guanzhu, Dianbai, Guangdong	Cultivated variety
CX127	Shadong, Guanzhu, Dianbai, Guangdong	Cultivated variety
CX128	Tangliaodi, Guanzhu, Dianbai, Guangdong	Cultivated variety
CX129	Jicai, Guanzhu, Dianbai, Guangdong	Cultivated variety
CX130	Jicai, Guanzhu, Dianbai, Guangdong	Cultivated variety

CX131	Jicai, Guanzhu, Dianbai, Guangdong	Cultivated variety
CX132	Jicai, Guanzhu, Dianbai, Guangdong	Cultivated variety
CX133	Jicai, Guanzhu, Dianbai, Guangdong	Cultivated variety
CX134	Jicai, Guanzhu, Dianbai, Guangdong	Cultivated variety
CX135	Pingshan, Guanzhu, Dianbai, Guangdong	Cultivated variety
CX136	Jicai, Guanzhu, Dianbai, Guangdong	Cultivated variety
CX137	Pingshan, Guanzhu, Dianbai, Guangdong	Cultivated variety
CX138	Shenwu, Shadong, Guanzhu, Dianbai, Guangdong	Cultivated variety
CX139	Tangliaodi, Guanzhu, Dianbai, Guangdong	Cultivated variety
CX140	Hedong, Guanzhu, Dianbai, Guangdong	Cultivated variety
CX141	Hedong, Guanzhu, Dianbai, Guangdong	Cultivated variety
CX142	Paizai Agarwood Mountain, Shalang, Dianbai, Guangdong	Cultivated variety
CX143	Shuijidong, Dianbai, Guangdong	Cultivated variety
CX144	Niutouhu, Guanzhu, Dianbai, Guangdong	Cultivated variety
CX145	Tangliaodi, Guanzhu, Dianbai, Guangdong	Cultivated variety
CX146	Shenwu, Shadong, Guanzhu, Dianbai, Guangdong	Cultivated variety
CX147	Shuijidong, Guanzhu, Dianbai, Guangdong	Cultivated variety
CX148	Xinpo, Haikou, Hainan	Wild variety
CX149	Xinpo, Haikou, Hainan	Wild variety
